# What Drives Knowledge Seeking, Sharing, and Use Among Family Planning Professionals? Behavioral Evidence From Africa, Asia, and the United States

**DOI:** 10.9745/GHSP-D-22-00036

**Published:** 2022-08-30

**Authors:** Ruwaida M. Salem, Anne Ballard Sara, Salim Seif Kombo, Sarah Hopwood, Tara M. Sullivan

**Affiliations:** a Johns Hopkins Center for Communication Programs, Baltimore, MD, USA.; b Busara Center for Behavioral Economics, Nairobi, Kenya.; c Busara Center for Behavioral Economics, Nairobi, Kenya; Now with Stanford Impact Labs, Stanford, CA, USA.

## Abstract

**Objective:**

To contribute to strengthening family planning and reproductive health (FP/RH) programs by identifying behavioral factors that influence FP/RH professionals’ knowledge management (KM) behaviors.

**Methods:**

We conducted an online survey, in-depth interviews, and cocreation workshops between July 2019 and June 2020 with a convenience sample of FP/RH professionals in Africa, Asia, and the United States to explore their KM behaviors. We used descriptive statistics to analyze the survey data and inductive thematic analysis for the interviews, and we synthesized participant inputs from selected cocreation activities.

**Results:**

The samples consisted of 273 survey respondents, 23 interviewees, and 69 cocreation workshop participants. There were no significant differences in how professionals seek and share information by gender, role, or geographic region, except related to language barriers among Francophone professionals. FP/RH professionals reported using both digital sources and their professional networks to seek and share information. Choice overload and cognitive overload (when people are presented with too much information and in a way that is hard to understand, respectively) act as barriers as they seek and use information. Too many information sources lead to frustration and inaction and best practices are often not contextualized or specific enough for application. Positive KM organizational cultures help facilitate effective information sharing, but reluctance to share information persists due to fear of losing comparative advantage. FP/RH professionals noted that such barriers result in duplication of effort and lack of advancement in FP/RH programs.

**Conclusion:**

To improve overall program impact, KM interventions in FP/RH and global health should reduce cognitive and choice overload, especially by curating and sharing practical, actionable information with essential details on context and how programs are implemented so that others can apply or adapt the learnings. Programs should use incentives to foster motivation to share this type of information.

## Abstract (precis)

To reduce the knowledge-to-action gap in global health programs, knowledge management (KM) interventions can apply behavioral economics concepts by sharing practical, actionable information on context and how programs are implemented, using a multifaceted KM approach to build trust and group identity among members, and using incentives to motivate information sharing.

## Teaser

**Key Findings:**

- Having too many information sources (choice overload) and too much information presented in a way that is difficult to understand (cognitive overload) are 2 barriers to seeking and using information for health care professionals.
- Developing innovative ways to empower individuals to curate their own centralized database or portal with timely and context-specific data may be a practical and effective way to mitigate these barriers.
- Positive knowledge management (KM) organizational cultures help facilitate effective information sharing within and across organizations. However, reluctance to share information persists due to fear of losing comparative advantage.

**Key Implications:**

- Family planning and global health should share practical, actionable information with important details on context and the “how” of program implementation so that others can apply or adapt the learnings more easily.
- Programs should use a mix of KM approaches to facilitate transparent dialogue and build trust, visibly recognize and provide feedback about the usefulness of shared information, and make it easy to share information through standard formats and familiar platforms and features.

## INTRODUCTION

Knowledge is an essential component of all 6 health systems' building blocks, including health service delivery and the health workforce.^1^ There is an urgent need for health care professionals worldwide to access, share, and apply evidence-based and experiential knowledge to enhance health systems, achieve health and development objectives, and, ultimately, improve people’s health and lives.

In addition, a broad range of global health stakeholders, from policy makers and program managers to researchers and practitioners, must collaborate and coordinate across organizations and geographic boundaries to ensure effective and efficient use of scarce resources. Recent infectious disease outbreaks, including Ebola and the COVID-19 pandemic, have highlighted the critical need for these stakeholders to harness and share knowledge effectively to make informed and timely decisions.^2^^–^^4^

Knowledge management (KM) is a discipline that integrates people, processes, and technology to collect and curate knowledge systematically and connect people to it so they can act effectively.^5^^–^^7^ It can help health care professionals access, share, and apply timely and relevant information. KM can also facilitate collaboration across professional, organizational, and geographic boundaries, expediting the global health field’s responsiveness to emergencies and health and development challenges.^3^^,^^8^

Within global health, voluntary family planning and reproductive health (FP/RH) programs have made substantial investments to translate research into practice to improve FP/RH services. For example, the United States Agency for International Development (USAID) has collaborated with the Johns Hopkins Center for Communication Programs (CCP) over several decades to share FP/RH and related global health knowledge around the world, through such seminal KM tools and guidelines as the *Family Planning High Impact Practices*, *Family Planning: A Global Handbook for Providers*, and more.^9^

Despite these achievements in KM for FP/RH, FP/RH professionals often do not fully engage in the cycle of knowledge seeking, sharing, and use. USAID and the Knowledge SUCCESS (Strengthening Use, Capacity, Collaboration, Exchange, Synthesis, and Sharing) project, primed by CCP, decided a new approach was needed to bridge this persistent knowledge-to-action gap and chose to apply behavioral economics—the study of economics and psychology to understand how and why people make decisions—to this challenge. Behavioral economics can help explain why individuals do not engage in these KM behaviors despite the added value, and it can also help design KM activities and solutions that mitigate barriers and are driven by the needs of health care professionals.

We used behavioral economics to bridge the persistent knowledge-to-action gap and help design KM solutions that were driven by the needs of health care professionals.

This article shares findings from research conducted by the Knowledge SUCCESS project to identify the behavioral factors that increase or hinder the likelihood of FP/RH professionals engaging in KM to improve their programs.

## METHODS

Between July 2019 and June 2020, we conducted an online survey, in-depth interviews, and participatory research through cocreation workshops, and applied a behavioral economics lens to analyze and triangulate the data to gain a deeper understanding of the current KM behaviors and needs of FP/RH professionals around the world. To be included in the research, individuals had to work in FP/RH programs and identify in such professional roles as a program manager, decision maker, technical advisor, or researcher. Data were collected and analyzed sequentially, starting with the online survey. Results from the online survey informed the focus of in-depth interviews, which then informed the design of the cocreation participatory research tools. This research received ethical approval from the Johns Hopkins School of Public Health Institutional Review Board.

### Online Survey

In August 2019, we conducted an online survey of global health professionals in English and French, drawing on a convenience sample of subscribers to global health and FP/RH listservs. The survey took approximately 15 minutes to complete and focused on identifying trends relating to demographic characteristics, KM behaviors, attitudes, and organizational culture. Analysis, mainly using descriptive statistics, was conducted in R statistical computing and graphics software (www.r-project.org).

### In-Depth Interviews

All survey respondents were subsequently given the option to participate in an in-depth interview, conducted in English or French, between August and November 2019. The 1-hour interviews were semi-structured, one-on-one conversations over Skype. The purpose of the interviews was to explore KM behaviors in more depth, including how FP/RH professionals seek, share, and use information and the barriers they face. All interviews were transcribed and analyzed using inductive thematic analysis.

### Participatory Research

Between April and June 2020, we conducted a series of cocreation workshops to reimagine the ways FP/RH professionals access and use evidence and best practices to optimize their programs. In cocreation workshops, designers convene a group of people who represent their audience—in this case, FP/RH professionals around the world—to bring them into the design process. In our cocreation workshops, we convened FP/RH professionals from government health offices, private entities, NGOs, and USAID across Africa, Asia, and the United States to design new KM solutions that address their needs. The Africa and Asia workshops were implemented over a 4-week period, with approximately 4 to 6 hours of working time per week. The U.S. workshop was conducted over 2 half-days. All sessions were conducted virtually through Zoom.

Using a design thinking process—a multidisciplinary, creative approach to problem solving that is iterative in nature and rooted in empathy with user needs—participants completed a number of individual and collaborative activities over 5 stages: empathize, define, ideate, prototype, and test. After the workshops, we analyzed the completed activities from the empathize and define stages to elicit insights on FP/RH professionals’ KM behaviors and challenges. These activities included KM profiles (or personas) of the participants’ KM experiences and a brainstorming activity to identify strengths, opportunities, and challenges related to accessing and using evidence and best practices.

### Triangulation Approach

We triangulated data from the online survey, in-depth interviews, and cocreation participatory research, with the online survey providing data on the methods used by FP/RH professionals when seeking and sharing information, and the in-depth interviews and cocreation research providing details on the barriers and opportunities FP/RH professionals face throughout their KM processes. We used a journey map to visualize the triangulated data to illustrate the general process, barriers, and opportunities that FP/RH professionals use when seeking and sharing information, with a common behavioral economics framework to organize the barriers and opportunities.

## RESULTS

### Background Characteristics

A total of 273 individuals from 52 countries met the inclusion criteria and completed the survey. The majority were men (63%) and were located in the Africa region (71%) (Table 1). We also conducted in-depth interviews with 23 FP/RH professionals from 14 countries, with a relatively even number of women and men. Most (n=19) were based in Africa. In the cocreation workshops, we convened 69 participants representing the United States and 20 countries in Africa and Asia, also with a relatively even split between women and men. Most of the survey and interview participants were program managers, researchers, or technical advisors while program managers and technical advisors made up the majority of workshop participants.

**TABLE 1.** Key Background Characteristics of Survey Respondents, Interview Respondents, and Workshop Participants to Explore Knowledge Management Behaviors Among Family Planning and Reproductive Health Professionals

	**Survey, No. (%) (N=273)**	**In-depth Interviews, No. (%) (N=23)**	**Workshop, No. (%) (N=69) **
Gender			
Men	172 (63.0)	13 (56.5)	35 (50.7)
Women	98 (35.9)	10 (43.5)	33 (47.8)
Non-binary	0 (0)	0 (0)	1 (1.4)
Prefer not to answer	3 (1.1)	0 (0)	0 (0)
Region			
Africa	193 (70.7)	19 (82.3)	36 (52.2)
Asia	44 (16.1)	1 (4.3)	20 (29.0)
North America	30 (11.0)	2 (8.7)	13 (18.8)
Europe	3 (1.1)	1 (4.3)	0 (0)
South America	3 (1.1)	0 (0)	0 (0)

From the survey, we did not find any statistically significant differences in the key KM behaviors of seeking and sharing information by gender, professional role, or geographic region. We also did not find major differences between these background characteristics and KM behaviors, challenges, and opportunities from the qualitative data (interviews and workshops) except as they related to language barriers, particularly among Francophone FP/RH professionals in sub-Saharan Africa. Therefore, we present descriptive results largely in the aggregate among all FP/RH professionals but note any substantive differences by region when relevant.

### How FP/RH Professionals Seek, Use, and Share Information

**FIGURE 1:**
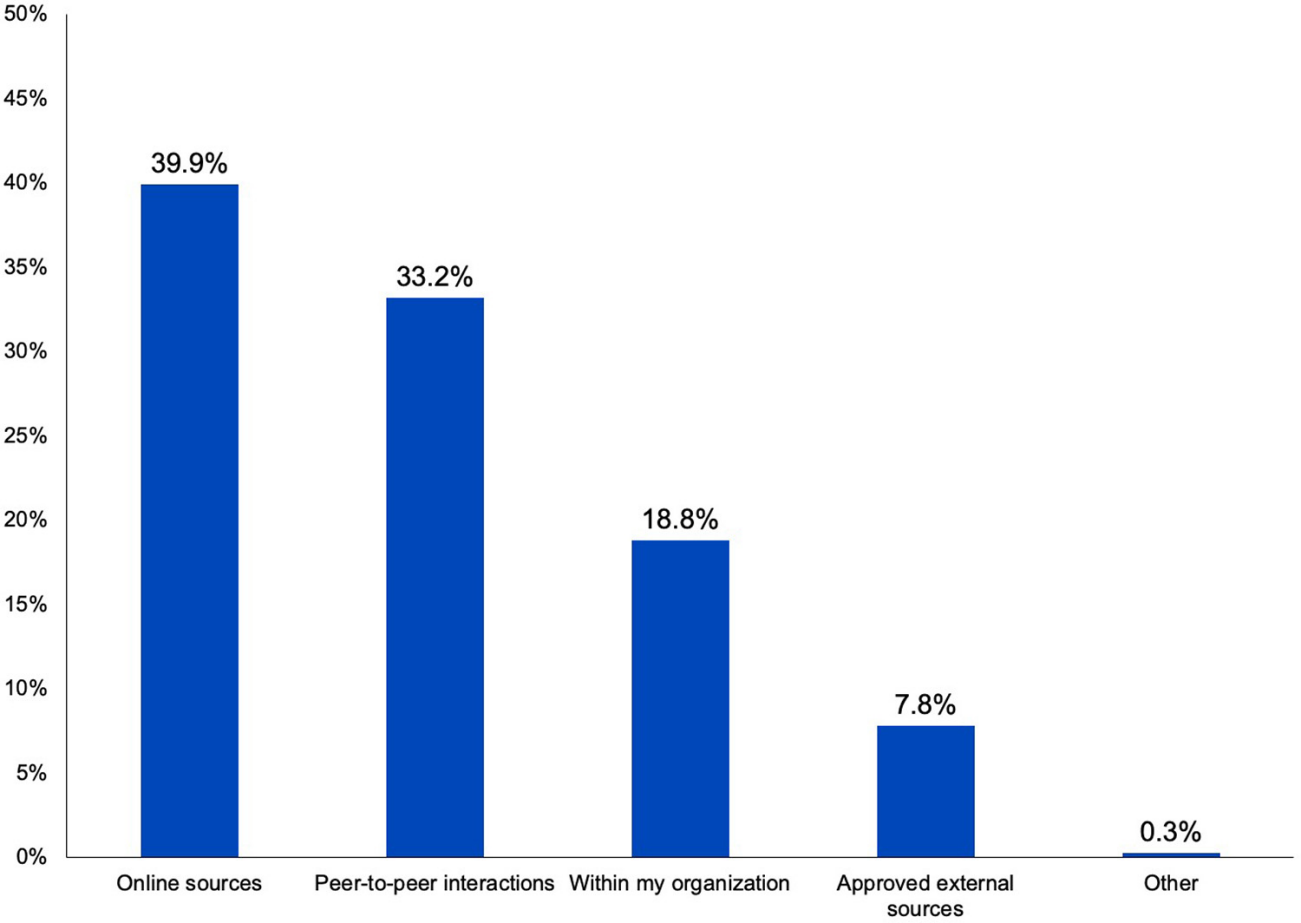
Most Commonly Used Methods by FP/RH Professionals to Find Information (N=273) Abbreviation: FP/RH, family planning and reproductive health.

**FIGURE 2:**
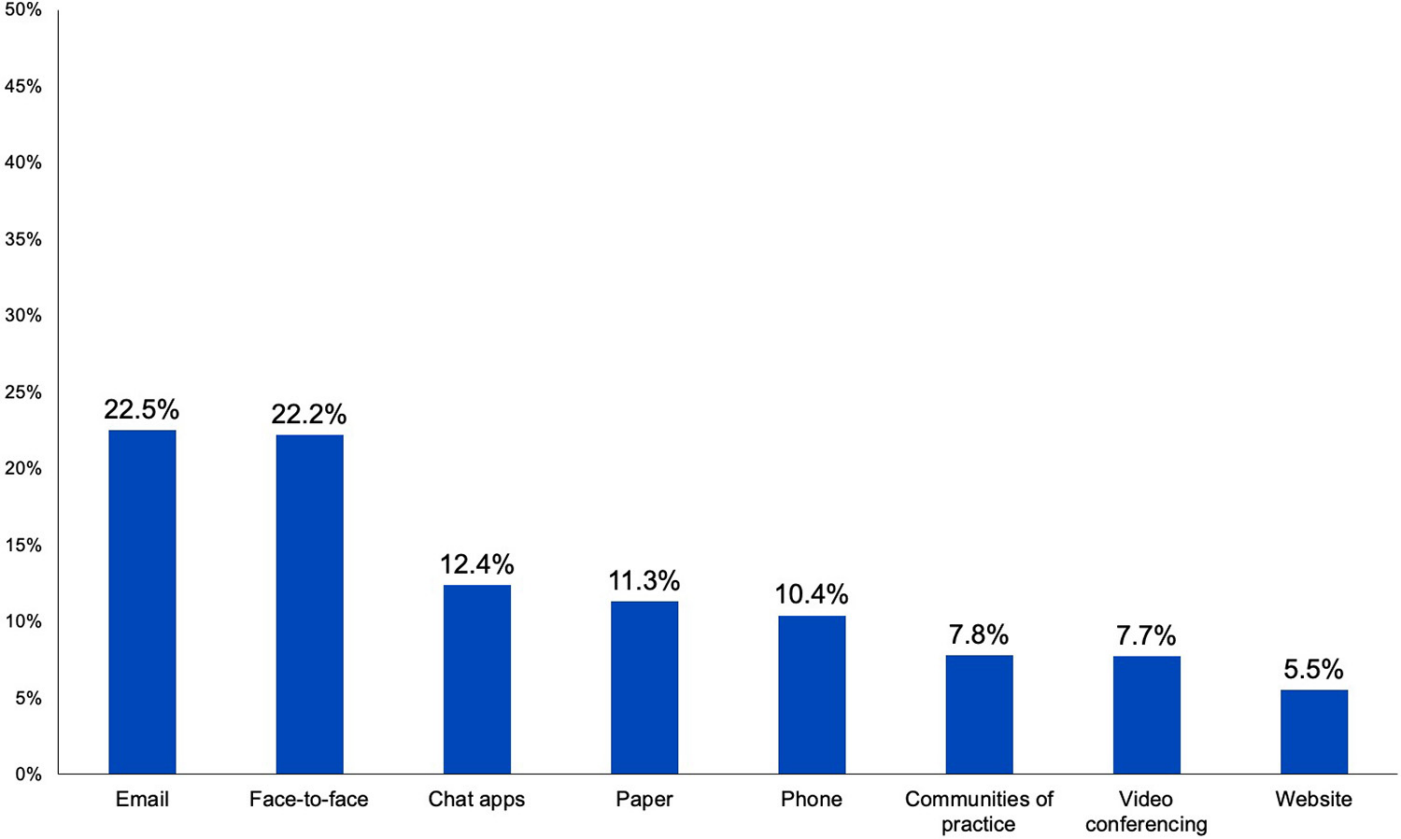
Most Commonly Used Methods by FP/RH Professionals to Share Information (N=273) Abbreviation: FP/RH, family planning and reproductive health.

FP/RH professionals in the survey, interviews, and workshops reported using both digital sources and their professional networks and colleagues to seek and share information. For example, survey respondents reported they most commonly seek information through online sources (39.9%) and peer-to-peer interactions (e.g., meetings and workshops) (33.2%) (Figure 1). Similarly, they most commonly reported using email (22.5%) and face-to-face interactions (22.2%) to share information (Figure 2). Chat apps (e.g., WhatsApp) and paper formats also featured prominently as methods for sharing information. It should be noted that all of our data collection methods relied on digital engagement (through online surveys, Skype-based interviews, or Zoom-based workshops), so participants’ use of digital information sources is expected, to some extent.

By triangulating both the quantitative and qualitative data from our research, we identified barriers that hinder FP/RH professionals’ ability to effectively seek, use, and share information and some important opportunities to facilitate information sharing, presented by their associated behavioral economics concepts. These concepts explain how people routinely and predictably deviate from decisions that might be in their (or others’) best interest. When applied in programs, the concepts can help identify which behaviors to encourage or mitigate to achieve program objectives, such as encouraging health care professionals to share critical knowledge with other professionals to reduce duplication of effort and avoid pitfalls.

**FIGURE 3:**
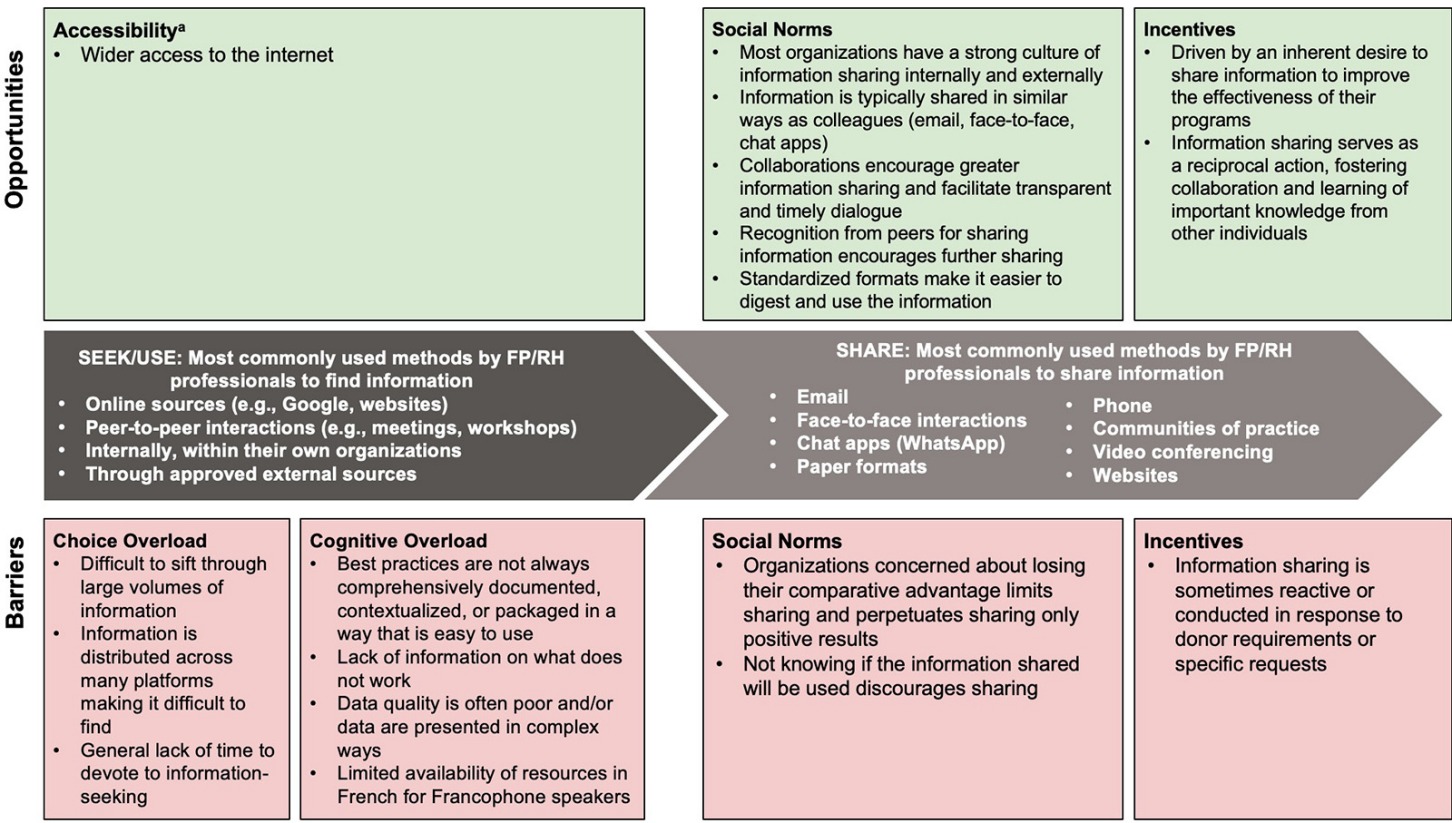
Key Behavioral Barriers and Opportunities Among FP/RH Professionals That Facilitate or Hinder Information Seeking, Use, and Sharing Abbreviation: FP/RH, family planning and reproductive health. ^a^ Accessibility is the ability to obtain information considering cost, format, language, timing, and technology.

We present the KM behaviors of seeking and using information together because similar behavioral concepts were identified, followed by behavioral barriers and opportunities to sharing information (Figure 3).

### Behavioral Barriers to Seeking and Using Information

We identified 2 key behavioral barriers—choice overload and cognitive overload—that impede FP/RH professionals’ ability to seek and use information effectively.

#### Choice Overload

Choice overload describes a situation when people are presented with too many choices, which can be mentally difficult to process, leading to frustration and inaction. When confronted with too many choices, people tend to go with the default option or to defer (put off) making a choice or taking action.

Choice overload—when people are confronted with too many choices—leads to frustration and inaction for FP/RH professionals.

FP/RH professionals indicated that it is easier to find information now than in the past with wider access to the internet but, at the same time, they are often overwhelmed by the sheer quantity of information.

*… there is so much information available from different sources. How to synthesize that information and to use it for your own purposes? …* —Workshop participant

When FP/RH professionals search for information, they feel overwhelmed because all of the information they need is seldom found in 1 platform, making it harder to sift through.

*It’s difficult and frustrating knowing there’s a whole host of information out there and it’s not all in 1 place.* —Interview respondent

#### Cognitive Overload

Cognitive overload describes a phenomenon when too much information is presented in a way that is hard to understand, making it difficult for people to process and apply the information.

Many interview respondents reported they need information that they can directly apply to make decisions about or solve problems in their programs, but information is often not contextualized or specific enough for their work. Workshop participants also reported that best practices are not always documented comprehensively, contextualized, or packaged in a way that is easy to use, limiting the utility of the information.

*Data is available on technical aspects of FP but when it comes to how to reach people and what's worked, that's where the data is limited or nonexistent.* —Workshop participant

Respondents reported that information is often not contextualized or specific enough for their work.

Workshop participants from Francophone Africa particularly noted language barriers in seeking and using information to inform their programs due to the limited availability of resources in French.

### Behavioral Barriers and Opportunities to Sharing Information

We identified 2 other behavioral concepts—social norms and incentives—that can act as either barriers to or opportunities for effective information sharing.

#### Social Norms

Social norms are the spoken or unspoken rules that create behavioral expectations for members of a group of people. In the case of KM for FP/RH and other health programs, these groups can be defined at the organizational or project level or more broadly within a community of practice or working group. Norms can cover a wide range of behaviors, including information sharing, signaling those behaviors as ones that are either encouraged or discouraged.

Three-quarters of the survey respondents indicated their organizations had positive KM cultures that encouraged information sharing. Furthermore, the most common reason reported for using the information sharing channel that they did was that it is what everyone else in their organization used, suggesting that individual sharing behavior is largely driven by organizational norms.

Interviewees and workshop participants noted that structured opportunities where sharing is an expectation, such as collaborations and partnerships, allowed for greater information sharing and learning. These opportunities help facilitate transparent and timely information sharing because those who share receive recognition and because sharing can happen through face-to-face dialogue rather than through formal, time-consuming documentation and dissemination approaches.

*There are so many formalities [to sharing information]. By the time they decide to disseminate data it’s already obsolete.* —Workshop participant

Additionally, FP/RH professionals noted that norms for reporting, such as the standardized format used in academic papers, help ensure sharing of robust information and make it easier to internalize and use the information.

Despite these positive norms, reluctance to share information persists in FP/RH due to competition among organizations for donor funding and concern about organizations losing their comparative advantage.

*If you give too much, others can take advantage of that.* —Interview respondent

Despite having positive information-sharing organizational cultures, respondents noted that reluctance to share information persists in FP/RH due to competition for funding and concern about losing comparative advantage.

Similarly, an organizational culture that perpetuates sharing only positive results to ensure continued funding hinders accountability and innovation.

*[There is] no direct sharing of what didn’t work—that norm is shifting a bit but it’s still an issue.* —Workshop participant

Many workshop participants pointed to the problems that this absence of sharing creates, including reinventing the wheel and a lack of growth and advancement of the field.

*We need to know what’s not working. We have successes but it's not pushing us to the next level.* —Workshop participant

Another commonly cited barrier to information sharing was not knowing if the information shared would be used.

*I always wonder what happens when I’m working on something like a policy brief. How is it used? What actually happens? … what’s taken away?* — Workshop participant

#### Incentives

Incentives are factors that motivate people to do something. Incentives can be intrinsic (an inner drive that propels a person to do something) or extrinsic (external factors that drive an individual to do something). Examples of extrinsic incentives include monetary and nonmonetary rewards, such as recognition or verbal praise for a job well done or a letter of appreciation.

For KM, incentives are most relevant when it comes to sharing information. Findings from the survey, interviews, and the workshops suggest that FP/RH professionals are driven by an inherent desire to share their knowledge with peers and colleagues to improve the effectiveness of their programs. This also serves as a reciprocal action, fostering collaboration with and learning from other individuals. However, data from the in-depth interviews and workshops suggest the behavior of sharing information also often appears to be reactive, conducted in response to donor or job requirements or when someone specifically asks for information.

*Donor requirements for making information public forces projects to put things on some sort of external site that might not have been required in the past.* —Workshop participant

While some level of information sharing is happening, often due to external requirements, the type of information FP/RH professionals typically share is formal—for example, a donor report or academic paper. This contributes to the difficulty of finding information that FP/RH professionals need to inform their work, including details on the “how” of program implementation and lessons learned from failures.

*Knowledge still dies with projects, especially the process knowledge.* —Workshop participant

## DISCUSSION

This research applies a behavioral economics lens to identify factors that facilitate and/or create barriers to positive KM behaviors among FP/RH professionals. Although the findings of this study may be familiar to KM experts in the FP/RH field, framing them with a behavioral economics lens provides new insights on how KM interventions can motivate health care professionals to bridge the knowledge-to-action gap. While the study focused on FP/RH professionals’ journey in KM, we think they are also likely applicable to the broader field of KM for global health. Table 2 summarizes how programs can apply select behavioral economics concepts to achieve key KM objectives to improve global health programs, with specific examples of how the Knowledge SUCCESS project has operationalized those ideas into its more recent activities and products for the global FP/RH community.

**TABLE 2.** Applying Behavioral Concepts to Knowledge Management Practice to Improve Programs

**Knowledge Management Objective**	**Promote or Mitigate Behavioral Economics Concept**	**Ways to Apply the Behavioral Economics Concept**	**Practical Examples from Knowledge SUCCESS Project**
Make it easy to find relevant and useful information	Mitigate choice overload	Curate information from various sources Provide more information sources for newer/emerging knowledge needs and fewer sources for established domains where the evidence and guidance have remained constant	20 Essential Resources (https://knowledgesuccess.org/20-essential-resources-2/) are collections of 20 essential resources on important FP/RH programmatic topics that are curated by a range of experts across organizations and projects That One Thing (https://knowledgesuccess.org/that-one-thing/): a weekly newsletter recommending the one tool, resource, or newsworthy item that FP/RH professionals should pay attention to that week FP insight (http://www.fpinsight.org/): a tool that allows individual FP/RH professionals to discover and curate their own collections of important resources
Use context-specific information to improve programs	Mitigate cognitive overload	Provide actionable information that includes enough detail on the “how” and the context Reduce dissemination of high-level “success stories” and instead include practical information on programming approaches that work **and** ones that don’t work	What Works in Family Planning and Reproductive Health (https://knowledgesuccess.org/2021/05/04/what-works-in-family-planning-and-reproductive-health-part-1-male-engagement/) is a series that draws on WHO’s guidelines for documenting program experiences and packages the details on the “how” of program implementation in an easily digestible and actionable way Learning Circles (https://knowledgesuccess.org/learning-circles/) are a small-group based and highly interactive series that guides program managers and technical advisors through supportive discussions on what works and what doesn’t in program implementation
Share information with other professionals within and across organizations	Promote social norms and internal incentives	Recognize individuals and organizations for sharing information in visible ways (e.g., badges, spotlights in newsletters)	FP insight users can earn badges (https://www.fpinsight.org/Badges) for completing certain actions on the platform, such as sharing resources, and they receive feedback, through notifications, when other users like their posts and follow their collections
		Make it easy to share information via templates and familiar features (e.g., social media icons) and formats (e.g., informal discussions) and provide feedback to those who share information	FP insight users can quickly and easily save and share resources (https://kmhelpdesk.knowledgesuccess.org/save-posts) to the platform by clicking on a visible and easily identifiable “plus” (+) icon
		Build trust and group identity among communities of practice by using a mix of interactive and online knowledge management tools and techniques	Learning Circle cohorts begin with various icebreakers to give participants an opportunity to build trust with each other, and they use a number of different KM tools and techniques (https://www.fpinsight.org/collection/618285f17050c200092934a4), such as 1-2-4-All and Troika Consulting, to facilitate information sharing in the interactive discussions Fail Fests encourage FP/RH professionals to reflect on program failures through small-group discussions with one member sharing a 2-minute failure story and the other members asking “curious questions” to reflect on lessons that can be applied in future activities

Abbreviations: FP/RH, family planning and reproductive health; KM, knowledge management; WHO, World Health Organization.

Because KM is a complex behavior, relying on just 1 approach—such as publishing fact sheets, briefs, and reports—will not result in optimal KM outcomes. Instead, the results from our study suggest that using a mix of KM tools and techniques, including conventional publications, databases, and websites as well as interactive events where people can connect and share tacit knowledge, will be more effective in making critical health information available and accessible, facilitating its use, and meeting health professionals where they are.

Results from our study suggest that using a mix of KM tools and techniques will yield more optimal KM outcomes than will relying on just 1 approach.

### How to Promote the Use of Evidence and Best Practices in Health Programs

Cognitive and choice overload are common—and related—barriers faced by health care professionals. Analyzing and determining which information sources are authoritative and provide relevant and useful information requires a considerable amount of time and effort, which are generally in short supply among busy health professionals. These findings support previous research from multiple countries where health care professionals expressed difficulty accessing comprehensive country-specific data and confusion about which sources were the most accurate.^10^ Such barriers can result in health care professionals falling back to the same sources of information and potentially using outdated information.

To address this problem, KM solutions for FP/RH have attempted to create “one-stop shops,” or centralized databases and portals, that curate information from various sources into 1 place. Although there is a benefit and need for such portals (e.g., the PubMed database is a clear example of a widely used and useful “one-stop shop” for scholarly biomedical and health literature), many of these collections quickly transform into a proliferation of information. That leaves the question of how many choices are too many. One possible solution is to provide more information sources for newer, emerging, or urgent technical areas, such as self-care interventions, universal health coverage, and ensuring essential FP/RH services during COVID-19, and fewer options for domains in which people are already knowledgeable and for which the evidence and guidance have remained constant. This is supported by previous research that found that decision makers with high subjective knowledge on a given topic were less willing to make a decision when choosing from a large set of options, whereas those with less knowledge preferred more options.^11^ In addition, the diversity of the global health field and its frequent dependence on context-specific data and solutions complicate the creation of 1 centralized source of information relevant to all health care professionals. Developing innovative ways to empower health care professionals to curate their own one-stop shops with minimal effort may be more practical and effective. One such approach that emerged from our cocreation workshops was an online platform called FP insight (www.fpinsight.org) that allows individual FP/RH professionals to discover and curate their own collections of important resources, taking inspiration from popular social media platforms such as Pinterest.

FP insight allows FP/RH professionals to discover and curate their own resource collections.

Our findings also emphasize the importance of ensuring that information produced is packaged in actionable, clear terms to promote information use—the major gap to overcome in KM for global health.^10^ This includes documenting program implementation in enough detail, including key information about context, to facilitate replication and adaptation of best practices among other professionals. Program stakeholders can refer to WHO guidelines and tools to ensure they report the design, implementation, monitoring, and evaluation of their programs more completely and accurately.^12^^,^^13^ Based on these guidelines, Knowledge SUCCESS has packaged program implementation details and lessons in easy-to-digest and interactive ways, such as through the What Works in Family Planning and Reproductive Health series.^14^ Increasing the documentation and sharing of such information while minimizing dissemination of high-level “success” stories that leave out important details of the “what” and “how” will help to reduce both cognitive and choice overload among health professionals and ultimately help avoid duplication of effort and missed opportunities in effective programming.

Packaging program implementation details and lessons in easy-to-digest ways will help reduce cognitive and choice overload.

### How to Motivate Health Care Professionals to Share Important Knowledge

We found that information-sharing behavior is largely driven by organizational or social norms, which is understandable since information sharing is a social behavior. The strong role of social norms means that instilling new behavior throughout an organization or a network provides the opportunity for widespread adoption, but conversely, a failure to do so risks its nonadoption.

Our findings suggest several types of interventions to foster positive social norms that encourage information sharing. The first is to recognize individuals and organizations for sharing useful information. FP/RH professionals value collaborations and partnerships for their information sharing opportunities and acknowledge that recognition for sharing information encourages further sharing. Using creative ways to provide more visible recognition to FP/RH professionals who share important information, such as through “badges” linked to a person’s profile or acknowledgment in a special section of a community newsletter for “champion” knowledge sharers, could help motivate people to increase their sharing. In addition, KM interventions should make it easier for people to share information by creating spaces for learning and sharing as part of established meetings, communities of practice, and conferences, using standard templates or platforms, formats, and features that are familiar to people, such as social media sharing icons, email communication, chat apps, and informal discussions. Furthermore, providing feedback to people whenever others use their contributions, such as verbal recognition or in the form of a usefulness rating scale, can help reinforce to people that the content they are sharing is in fact useful and will be used by others. Finally, our findings support the use of a multifaceted KM approach that includes interactive techniques via meetings and communities of practice, to build trust and group identity among members, in addition to online platforms and tools to help reinforce the social norms of robust information sharing. This aligns with a research review that recommended several ways to encourage group members to cooperate and share information, including increasing the perceived benefits and efficacy of contributing to knowledge exchange, reducing the perceived costs, and establishing group identity.^15^

Our findings support use of a multifaceted KM approach including interactive techniques with online platforms and tools.

### Limitations

A notable limitation of this research was the use of convenience sampling for the survey, in-depth interviews, and participatory research. Therefore, caution should be used in generalizing the findings to all FP/RH professionals. However, by triangulating data across the 3 data sources, we have provided a deeper understanding of FP/RH professionals’ KM behaviors, barriers, and opportunities.

## CONCLUSION

This research provides in-depth understanding of the current KM behaviors, motivations, and needs of FP/RH professionals around the world and has broad implications for how to improve KM effectiveness and efficiency in FP/RH programs and the wider global health field. In particular, KM solutions should reduce cognitive and choice overload, especially by sharing practical, actionable information with important details on context and how programs are implemented so that others can apply or adapt the learnings. Fostering motivation to share this type of information can improve dynamism in KM and overall impact in FP/RH programs. The insights from this research have shaped the focus of new KM solutions developed by the Knowledge SUCCESS project, including FP insight, a user-driven resource curation and discovery platform, and Learning Circles, an interactive learning series focused on the details of what works and what doesn’t in FP/RH programs.^16^ The findings also have the potential to benefit and inform the KM approaches and practices of other global health projects and organizations.

## Notes

**Peer Reviewed**

**First published online:** August 3, 2022.
